# Molecular Analysis of Sarcoidosis Tissues for **Mycobacterium** Species DNA

**DOI:** 10.3201/eid0811.020318

**Published:** 2002-11

**Authors:** Wonder Puryear Drake, Zhiheng Pei, David T. Pride, Robert D. Collins, Timothy L. Cover, Martin J. Blaser

**Affiliations:** * Vanderbilt University School of Medicine and Veterans Affairs Medical Center, Nashville, Tennessee, USA; † New York University School of Medicine and Veterans Affairs Medical Center, New York, New York, USA

**Keywords:** sarcoidosis, polymerase chain reaction, *Mycobacterium*, 16S rRNA, *rpoB*, IS*6110*

## Abstract

We performed polymerase chain reaction analysis, for *Mycobacterium* species 16S rRNA, *rpoB*, and IS*6110* sequences, on 25 tissue specimens from patients with sarcoidosis and on 25 control tissue specimens consisting of mediastinal or cervical lymph nodes and lung biopsies. *Mycobacterium* species 16S rRNA sequences were amplified from 12 (48%) *rpoB* sequences from 6 (24%) of the sarcoidosis specimens. In total, 16S rRNA or *rpoB* sequences were amplified from 15 sarcoidosis specimens (60%) but were not detected in any of the control tissues (p=0.00002, Chi square). In three specimens, the sequences resembled *Mycobacterium* species other than *M. tuberculosis*. All specimens with sequences consistent with *M. tuberculosis* were negative for IS*6110*. We provide evidence that one of a variety of *Mycobacterium* species*,* especially organisms resembling *M. tuberculosis,* is found in most patients with sarcoidosis.

Sarcoidosis is a multisystem inflammatory disease that mainly affects lymph nodes and pulmonary tissues and is characterized by noncaseating granulomata in affected organs (1). Although the cause of sarcoidosis remains unknown, several microorganisms have been proposed as possible etiologic agents, including bacteria (*Borrelia burgdorferi, Proprionibacterium acnes,* and *Mycobacterium* species) and viruses (*Human herpesvirus* 8, Epstein-Barr virus, *Cytomegalovirus*, and Coxsackie B) (2). Metals (beryllium and zirconium), minerals (talc and clay), and organic substances (pine tree pollen) have also been proposed as etiologic agents (2). Efforts to identify an infectious agent for sarcoidosis using methods such as histologic staining and routine microbial culture have been unsuccessful.

Polymerase chain reaction (PCR) analysis for microbial DNA serves as an alternative method for identifying infectious agents. PCR was used to identify the etiologic agents of bacillary angiomatosis (*Bartonella henselae)* (3) and Whipple’s disease (*Tropheryma whippelii)* (4). Because of the substantial pathologic (5), immunologic (6), epidemiologic (7), and clinical similarities (8,9) between sarcoidosis and infections caused by *Mycobacterium* species (particularly tuberculosis), we analyzed tissue specimens from patients with sarcoidosis for evidence of mycobacterial genes. The results of previous studies have been inconclusive; some investigators were unable to demonstrate mycobacterial DNA in sarcoid lesions (10,11), whereas others have amplified mycobacterial DNA of different species (12,13). We examined sarcoidosis and control paraffin-embedded pulmonary, mediastinal, and cervical tissue specimens for *Mycobacterium* species 16S rRNA, *rpoB*, and IS*6110* sequences.

## Materials and Methods

### Patients and Samples

For this study, we selected paraffin-embedded tissue specimens from patients who had had mediastinal or cervical lymph node resection from 1991 to 2001. Specimens from 44 patients with sarcoidosis and 57 controls were included. Patients were included for further study if they met the pathologic and clinical features described and if the specimens, after processing and DNA extraction, were positive for human β-actin with PCR analysis. We were unable to retrieve purified protein derivative status on a systematic basis**.** Based on these criteria, 25 control and 25 sarcoidosis specimens were further analyzed.

For inclusion in this study, the following criteria was used for patients with sarcoidosis: 1) clinical features had to be consistent with sarcoidosis (i.e., acute respiratory illness accompanied by erythema nodosum, hilar adenopathy and arthritis [Lofgren’s syndrome], or indolent progressive pulmonary decompensation associated with radiographic findings, such as hilar adenopathy, reticulonodular infiltrates, or pulmonary fibrosis); 2) histopathologic features had to be consistent with sarcoidosis (i.e., specimens from each patient had to have confluent noncaseating granulomas, well circumscribed within the surrounding tissue with a variable amount of peripheral lymphocytic infiltration [5]); 3) known microbial causes for granulomata had to be excluded (i.e., specimens were negative for microorganisms by hematoxylin and eosin (H&E), fungal, acid fast bacilli (AFB), and auramine-O stains and on routine bacterial, fungal, and AFB cultures). In each case, histopathologic specimens were independently reviewed by two pathologists.

Control lymph node specimens were selected from patients who had undergone mediastinoscopy or cervical node biopsy during the same period. In each case, a definitive diagnosis other than sarcoidosis was made. Control patients were selected from patients for whom the final diagnoses were fungal infection, lymphoma, and primary or metastatic lung malignancies (Table 1).

**Table 1:** Demographic and pathologic information for sarcoidosis and control tissue specimens^a^

Patient	Age (yrs)/sex	Race	Source of specimen	Pathologic diagnosis	Presence of granuloma
1	61/M	C	Lung	Sarcoidosis	Yes
2	60/F	C	Cervical node	Sarcoidosis	Yes
3	43/F	C	Mediastinum	Sarcoidosis	Yes
4	55/F	C	Mediastinum	Sarcoidosis	Yes
5	42/M	AA	Mediastinum	Sarcoidosis	Yes
6	54/F	C	Mediastinum	Sarcoidosis	Yes
7	48/F	C	Mediastinum	Sarcoidosis	Yes
8	34/M	C	Mediastinum	Sarcoidosis	Yes
9	72/F	AA	Mediastinum	Sarcoidosis	Yes
10	42/F	C	Mediastinum	Sarcoidosis	Yes
11	42/F	C	Mediastinum	Sarcoidosis	Yes
12	68/F	C	Mediastinum	Sarcoidosis	Yes
13	37/F	C	Mediastinum	Sarcoidosis	Yes
14	45/F	C	Mediastinum	Sarcoidosis	Yes
15	46/F	C	Mediastinum	Sarcoidosis	Yes
16	38/M	C	Mediastinum	Sarcoidosis	Yes
17	33/F	C	Mediastinum	Sarcoidosis	Yes
18	26/M	C	Mediastinum	Sarcoidosis	Yes
19	55/F	AA	Mediastinum	Sarcoidosis	Yes
20	31/M	C	Mediastinum	Sarcoidosis	Yes
21	42/M	C	Lung	Sarcoidosis	Yes
22	42/F	C	Mediastinum	Sarcoidosis	Yes
23	38/M	C	Mediastinum	Sarcoidosis	Yes
24	54/F	C	Mediastinum	Sarcoidosis	Yes
25	78/M	C	Mediastinum	Sarcoidosis	Yes
26	33/M	AA	Lung	Hodgkin’s disease	No
27	70/F	C	Lung	Histoplasmosis	Yes
28	73/M	C	Mediastinum	Mesothelioma	No
29	56/F	C	Mediastinum	Adenocarcinoma	No
30	24/M	C	Lung	Cryptococcus	Yes
31	75/M	C	Lung	Renal cell cancer	No
32	32/M	AA	Mediastinum	Coccidiomycosis	Yes
33	41/F	C	Mediastinum	Breast cancer	No
34	74/F	C	Mediastinum	Adenocarcinoma	No
35	72/F	C	Mediastinum	Large-cell cancer	No
36	77/M	C	Mediastinum	Large-cell cancer	No
37	72/M	AA	Mediastinum	Large-cell cancer	Yes
38	78/F	C	Mediastinum	Adenocarcinoma	No
39	72/M	C	Mediastinum	Squamous cell cancer	No
40	52/F	AA	Mediastinum	Adenocarcinoma	Yes
41	52/F	C	Mediastinum	Breast cancer	Yes
42	73/M	C	Mediastinum	Adenocarcinoma	Yes
43	18/M	AA	Mediastinum	Histoplasmosis	No
44	47/M	C	Mediastinum	Hodgkin’s lymphoma	No
45	74/M	C	Mediastinum	Large-cell lymphoma	No
46	76/M	C	Mediastinum	Adenocarcinoma	No
47	75/M	C	Mediastinum	Small-cell cancer	No
48	60/M	C	Mediastinum	Adenocarcinoma	No
49	73/M	C	Mediastinum	Adenocarcinoma	No
50	85/M	C	Mediastinum	Lymphoma	No
51	40/M	AA	Ileum	Tuberculosis	No

^a^F, female, M, male; C, Caucasian; AA, African-American.

### DNA Extraction

For each patient enrolled in the study, the original paraffin-embedded tissue block was retrieved from the archives, and eleven 10-µm sections were cut from each. One section was stained with H&E for microscopic examination, four sections were used for extraction of DNA, and the remaining six sections were stored for future analysis. The specimens were randomly processed for slide preparation, and the microtome blade was changed between each tissue block. For each section from patients with sarcoidosis and for the control specimens with the granulomata, granulomata were microdissected and extracted by using disposable surgical blades. For those control specimens without granulomata, all tissue from the four sections was used for DNA extraction. For all specimens, DNA was extracted with the Qiagen DNAeasy extraction kit (Qiagen, Valencia, CA) according to the manufacturer’s instructions, except that 60 µL of proteinase K was used at a concentration of 20 mg/mL. Tissue dissection and DNA preparation were performed in a dedicated clean room, which was separate from the rooms used for PCR analysis and sequencing. The extracted DNA was stored at –20°C. Groups of tissue specimens from patients with sarcoidosis and controls were processed in parallel during all steps of the procedure, including extraction of the DNA, amplification and detection of mycobacterial DNA, and sequence analysis.

### PCR Analysis for 16S rRNA, *rpoB*, and IS*6110*

Before PCR amplification, to ensure that the extracted DNA was of proper quality, we used PCR to verify that DNA sequences encoding human β-actin could be amplified. The primers used were 5´ATCATGTTTGAGACCTTCAAC3´ (forward primer) and 5´CAGGAAGGAAGGCTGGAAGAG3´ (reverse primer). The PCR conditions were 35 cycles of amplification carried out in a DNA Thermal Cycler 480 (Perkin-Elmer, Wellesley, MA); each cycle consisted of 1 min of denaturing at 94°C, 1 min of annealing at 54°C, and 1 min of extension at 72°C (14). As required, all 50 tissue specimens yielded human β-actin amplicons and were tested further for the presence of bacterial DNA.

For amplification of 16S rRNA sequences, a nested PCR analysis was performed. The primers FO16S, 5´GATAAGCCTGGGAAACTGGGTC3´ and RO16S, 5´TTCTCCACCTACCGTCAATCCG3´ were selected to amplify a 344-bp fragment of the 5´ region (nt 134–477) of mycobacterial 16S rRNA. Primers FI16S (5´CATGTCTTGTGGTGGAAAGCG3´) and RI16S (5´TACCGTCAATCCGAGAGAACCC3´) were selected as nested primers to amplify a 288-bp fragment (nt 181–468). The PCR conditions for both sets of primers were as follows: 5 min of denaturing at 94°C, followed by 35 cycles of amplification, consisting of 1 min of denaturing at 94°C, 1 min of annealing at 58°C, and 1 min of extension at 72°C. At the end of the 35 cycles, a final extension cycle of seven minutes at 72°C was performed.

For amplification of *rpoB* sequences, a nested PCR also was performed. The primers FOrpoB (5´GCAGACGCTGTTGGAAAACTTG3´) and ROrpoB, (5´TGTTCTGGTCCATGAATTGGCTC3´) were selected to amplify a 455-bp fragment of the β subunit (nt 1,940–2,394) of the *M. tuberculosis* RNA polymerase gene. The inner primers were designed as previously described and used in a nested fashion (nt 1,965–2,324) to amplify a 360-bp product (15). The PCR conditions were as described previously for 16S rRNA.

For amplification of IS*6110* sequences, PCR analysis included the use of primers IS*1* (5´CCTGCGAGCGTAGGCGTCGG3´) and IS*2* (5´CTCGTCCAGCGCCGCTTCGG3´), designed to amplify a 123-bp fragment (nt 1,510–1,632) of the *M. tuberculosis* IS*6110* element (16). The assay used the same conditions as described previously, with the exception that the PCR analysis included 30 rather than 25 cycles.

Negative and positive controls were run in parallel with each PCR assay. We used genomic DNA extracted from *M. tuberculosis* strain H37rv as positive controls, and DNA extracted from a paraffin-embedded tissue biopsy from an AIDS patient with ileocecal tuberculosis. We included the following as negative controls in each PCR reaction: DNA extracted from nonsarcoid paraffin-embedded tissue, PCR master mix inoculated with 5 µL of sterile water, and PCR master mix alone. The DNA extraction was performed in the same manner as described for the sarcoid and control specimens.

### Determination of DNA Sequence of Amplified Products

The PCR products were purified with the Qiagen QIAquick PCR purification kit (Qiagen, Valencia, CA) and sequenced directly on both strands in the Vanderbilt Cancer Center Core Sequencing Laboratory. In cases in which the signal was ambiguous, PCR products were cloned into the plasmid vector system, pGEM T-Easy (Promega, Madison, WI), and the nucleotide sequences were then determined.

Alignments of the 16S rRNA, *rpoB*, and IS*6110* sequences were performed with the NCBI BLAST program. Statistical evaluation of significance was determined by using Chi-square analysis or Fisher’s exact test, depending upon anticipated cell size. Sequences were aligned with ClustalW and subjected to phylogenetic analysis with HKY85 distance matrices with Paup 4.0b8 (Sinauer Associates, Sunderland, MA).

## Results

### Patient and Specimen Characteristics

Of the 25 patients with sarcoidosis, 12% were African American and 88% Caucasian; 36% were men, and 64% were <50 years of age. No specimens were obtained from persons <18 years of age (Table 1). The control population was 20% African American and 80% Caucasian; 68% were men. Most (76%) of the control patients were >50 years of age; the age and sex of the control patients reflect the patient population undergoing mediastinoscopy to obtain a tissue diagnosis for probable malignancy. The control population consisted of patients with lung cancer (72%), chronic fungal infections (16%), or lymphoma (12%) (Table 1). Mediastinal lymph nodes were the source of specimens from 88% and 84% of the sarcoid and control patients, respectively. The remaining specimens from each group were obtained from either pulmonary or cervical lymph node biopsies. Granulomas were present in all of the sarcoidosis tissue specimens and in 7 of the 25 control specimens.

### PCR Assay Sensitivities

The sensitivity of the PCR assay for each gene was determined by PCR analysis of serially diluted genomic DNA from *M. tuberculosis* strain H37rv, ranging from 5 ng to 0.05 fg per µL. One *M. tuberculosis* genome is estimated to have a mass of 5 fg (17). For PCR analyses of 16S rRNA, *rpoB,* and IS*6110*, we consistently achieved a sensitivity of 1–2 gene copies in each assay.

### 16S rRNA PCR of Tissue Specimens

In the PCR for 16S rRNA sequences (Table 2), 12 (48%) of the 25 sarcoidosis specimens tested positive compared to none of 25 of the control specimens (p=0.0003, Chi square). Sequence analysis of the PCR products from the sarcoidosis specimens showed that 8 of the 12 had 100% positional identity with *M. tuberculosis*, and 1 possessed 99% positional identity with *M. tuberculosis* (patient 15). Sequencing of the 16S PCR product of patient 15 showed a C®T substitution at position 289 and an A®G substitution at position 355 (based on the *M. tuberculosis* 16S rRNA sequence, GenBank accession nos. Z83862.1, AJ131120.1, X52917.1, and X58890.1). Three other sequences were found (in patients 7, 19, and 24) that most closely resembled other *Mycobacterium* species*.* The amplicon sequence from patient 7 possessed an A®G substitution at position 299 and a C®A substitution at position 380, yielding 99% positional identity with *M. kansasii.* Notably the sequences of *M. kansasii,* *M. avium, M. visibilis,* and *M. paratuberculosis* are identical within this region; therefore, distinguishing between these species is not possible (18). The amplicon sequence from patient 19 contained a T®C substitution at position 434, yielding 99% positional identity with *M. gordonae.* The amplicon sequence from patient 24 contained 100% positional identity with *M. gordonae* and *M. bohemicum* (19). The phylogenetic relationships of the mycobacterial sequences are shown in the Figure and are deposited in GenBank (accession nos. AF468214, AF468215, and AF468216).

**Table 2:** Analysis of sarcoidosis and control tissue specimens for *Mycobacteria* 16SrRNA, *rpoB*, and *IS6110*^a^

Sarcoidosis patient	16S rRNA	*rpoB*	IS*6110*	Control patient	16S rRNA	*rpoB*	IS*6110*
1	*M. tuberculosis*	*M. tuberculosis*	—	26	—	—	—
2	*M. tuberculosis*	—	—	27	—	—	—
3	—	*M. tuberculosis*	—	28	—	—	—
4	*M. tuberculosis*	—	—	29	—	—	—
5	*M. tuberculosis*	—	—	30	—	—	—
6	—	—	—	31	—	—	—
7	*M. kansasii*	—	—	32	—	—	—
8	—	*M. tuberculosis*	—	33	—	—	—
9	*M. tuberculosis*	—	—	34	—	—	—
10	—	—	—	35	—	—	—
11	*M. tuberculosis*	—	—	36	—	—	—
12	—	—	—	37	—	—	—
13	—	—	—	38	—	—	—
14	*M. tuberculosis*	*M. tuberculosis*	—	39	—	—	—
15	NM	—	—	40	—	—	—
16	—	*M. tuberculosis*	—	41	—	—	—
17	—	—	—	42	—	—	—
18	*M. tuberculosis*	—	—	43	—	—	—
19	*M. gordonae*	—	—	44	—	—	—
20	—	—	—	45	—	—	—
21	—	—	—	46	—	—	—
22	—	—	—	47	—	—	—
23	—	—	—	48	—	—	—
24	*M. gordonae*	*M. tuberculosis*	—	49	—	—	—
25	—	—	—	50	—	—	—
				51	*M. tuberculosis*	*M. tuberculosis*	*M. tuberculosis*
				H37rv	*M. tuberculosis*	*M. tuberculosis*	*M. tuberculosis*

^a^NM, novel mycobacterium, resembling *M. tuberculosis*; H37rv, *M. tuberculosis*; —, indicates a negative result.

**Figure:**
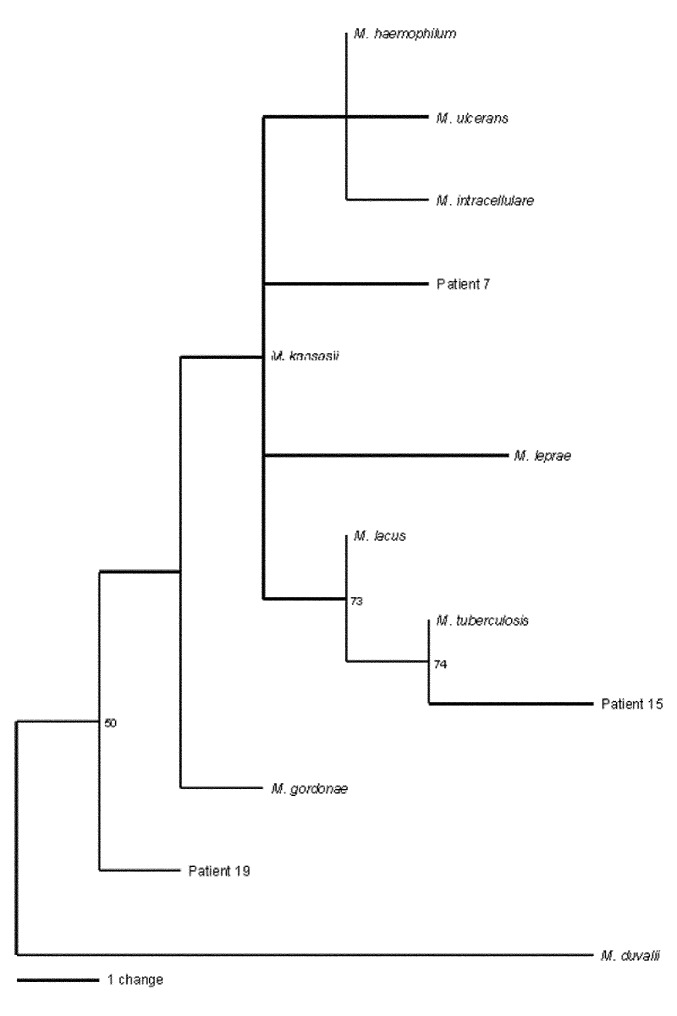
Analysis of 16S rRNA sequences from nine *Mycobacterium* species and from 12 patients with sarcoidosis. Phylograms based on nucleotide alignments were generated with HKY85 distances matrices using Paup 4.0b10 (Sinauer Associates, Sunderland MA). Bootstrap values >50 (based on 500 replicates) are represented at each node, and the branch length index is represented below the phylogram. For eight patients, the 16S rRNA sequence was identical to *M. tuberculosis*, and for one patient (patient 24) was identical to *M. gordonae*. For patient 15, the sequence was closely related to *M. tuberculosis*; for patient 19, the sequence was closely related to *M. duvalii* and *M. gordonae*; for patient 7, the sequence was less closely related to *M. leprae*.

### PCR of Tissue Specimens with Other Mycobacterial Primers

With the *rpoB* PCR, 6 (24%) of the 25 specimens from sarcoidosis patients yielded a product of 360 bp, which by sequence analysis in each case was most consistent with *M. tuberculosis.* Five sequences amplified from sarcoidosis specimens had 98%–100% positional identity with *M. tuberculosis* (patients 1, 3, 8, 14, and 24), whereas one had 95% positional identity (patient 16) (Table 2). The difference in the rate of finding *M. tuberculosis* *rpoB* sequences (24% in the sarcoidosis specimens and none in the control specimens) was also significant (p=0.02, Fisher’s exact test). In total, 15 (60%) of the sarcoidosis specimens had either *Mycobacterium* 16S rRNA or *rpoB* sequences compared with none of the control specimens (p=0.00002, Chi square). Mycobacterial 16S rRNA and *rpoB* fragments were amplified from 3 (12%) of the 25 sarcoidosis specimens (patients 1, 14, and 24). Patients 1 and 14 possessed sequences that had 100% positional identity with *M. tuberculosis* 16S rRNA and 98%–99% positional identity with *M. tuberculosis rpoB* DNA. The products amplified from patient 24 possessed 100% positional identity with *M. gordonae* 16S rRNA and 99% positional identity with *M. tuberculosis rpoB* DNA. In the region amplified with our 16S primers, a difference of 14 nt existed between the 16S rRNA of *M. tuberculosis* and *M. gordonae.* In the region amplified by using *rpoB* primers, a difference of 39 nt existed between *rpoB* of *M. tuberculosis* and *M. gordonae.*

None of the sarcoidosis or control patient tissue specimens yielded IS*6110* amplicons (Table 2). In contrast, IS*6110* products were consistently found in the positive controls, genomic DNA from *M. tuberculosis* H37rv, and DNA extracted from a paraffin-embedded tissue biopsy of an AIDS patient with ileocecal tuberculosis. Both positive controls were positive in the 16S rRNA, *rpoB*, and IS*6110* PCR assays, and sequence analysis of the products indicated 100% homology with *M. tuberculosis.* The negative controls were consistently negative.

## Discussion

For this study, we chose patients whose cases were consistent with sarcoidosis or in whom an alternative diagnosis was made conclusively. We chose this stringent design so that no borderline tissues were examined. Many cases of disease eventually diagnosed as sarcoidosis have atypical findings. If our present observations are confirmed, such cases will be important for future analyses.

We found evidence of mycobacterial DNA in the granulomas of 24% of sarcoidosis specimens when assessing for *rpoB,* 48% in the same population when assessing for 16S rRNA; in total, 60% were positive for either. We acknowledge the limitations of studying archival tissue and the possibility of contamination; however, control tissues did not demonstrate positive results, making contamination less likely. Earlier studies have identified the presence of mycobacterial DNA in sarcoidosis tissue specimens with 30%–50% prevalence (12,13,20). Instead of a single organism being present, we provide evidence for a heterogenous population of *Mycobacterium* species in the sarcoidosis tissue specimens studied. Although we found evidence of organisms resembling *M. tuberculosis, M. gordonae*, and *M. kansasii,* other studies also have identified *M. avium* sequences (12,13)*.*

We also provide DNA sequence evidence for novel mycobacteria in patient 15. Although most DNA sequences from the study patients most closely resemble *M. tuberculosis,* sequences resembling other mycobacterial species also were identified (Table 2, Figure). In several previous studies, non-tuberculosis mycobacteria also have been reported (13). One novel sequence is most closely related to 16S rDNA from *M. tuberculosis,* a known pulmonary pathogen, rather than to sequences from other mycobacterial species of lesser virulence. The consistent presence of two single polymorphisms in the same location in the novel sequence suggests a true polymorphism rather than an error introduced by Taq PCR. Moreover, the novel sequence was consistently absent from water, non-sarcoid paraffin-embedded tissue, and *M. tuberculosis* DNA controls. Synonymous substitutions in the *M. tuberculosis* genome are relatively rare, although genomic variations have been found in genes associated with antibiotic resistance (21). The DNA with the polymorphism suggests that a variant of *M. tuberculosis*, or a closely related novel mycobacterium, may be present in the sarcoidosis specimen.

The presence of *M. tuberculosis* DNA in 48% of sarcoidosis specimens is notable because clear clinical connections between sarcoidosis and tuberculosis have been made. On occasion, patients with documented tuberculosis develop sarcoidosis while on antituberculous treatment or vice versa (22–24). Mycobacterial DNA in sarcoidosis specimens may explain the clinical correlation between sarcoidosis and tuberculosis. That patients have developed sarcoidosis while on antituberculous therapy suggests that in those patients M. tuberculosis was not the etiologic agent of sarcoidosis. That 60% of the specimens we examined showed mycobacterial DNA agrees with certain previous studies (12,13), but other studies were negative for mycobacterial DNA (10,11). One possible explanation for these discordant results is that sarcoidosis represents one group of host responses to infectious agents of which mycobacteria represent the largest associated group. Alternatively, Mycobacterium species are present in many of the lesions but at extremely low levels, on either side of the threshold of detection. Such a hypothesis of small numbers of organisms provoking an intense inflammatory response, analogous to tuberculoid leprosy (25), could explain why organisms cannot be detected except by ultrasensitive methods. Yet another alternative explanation was our observation of degradation of the mycobacterial signal in the total DNA extract. We observed that mycobacterial DNA could be amplified from the positive specimens consistently over a 6–8 month period if the original DNA extract was maintained at –20oC. After this time period, fresh DNA extractions were necessary to demonstrate the presence of mycobacterial DNA. The original specimens, in which the mycobacterial DNA could no longer be amplified, remained positive for human β-actin by PCR analysis, although the band was weaker, suggesting either that the eukaryotic DNA degraded more slowly than prokaryotic DNA or that more signal was originally present. This degradation occurred despite minimizing freeze-thaws of extracted DNA and maintaining the DNA at –20°C. Our observation suggests that isolation of mycobacterial DNA from sarcoidosis specimens is best achieved by performing PCR analysis on fresh DNA extractions, which may help explain why other investigators had negative findings.

Based on these observations, we examined whether M. tuberculosis DNA was present in the sarcoid granuloma by testing for the presence of IS6110. PCR analysis for IS6110 is useful, since IS6110 is typically present in 1–25 copies in members of the M. tuberculosis complex. M. bovis BCG has only a single of copy of IS6110, whereas the higher copy numbers are typically found in M. tuberculosis isolates (26). We found no evidence of IS6110 DNA in our sarcoidosis or control tissue specimens.

Several possible explanations exist for the presence of mycobacterial 16S rRNA and rpoB, and the absence of IS6110 in the sarcoid specimens, although these three amplicons were consistently present in our positive controls. First, our assay for IS6110 may not have been sufficiently sensitive to detect the very low numbers of M. tuberculosis genomes in the sarcoidosis tissue specimens. In serial dilution studies, the assay was sensitive enough to detect one bacterial genome, comparable to results for the nested PCRs for 16S and rpoB. However, correlating the sensitivity of DNA extracted from bacterial culture to DNA extracted from formalin-fixed, paraffin-embedded tissue is not possible. Other laboratories that reported an assay sensitivity of 1–2 genome copies for IS6110 in sarcoidosis tissue extract were also unable to detect any IS6110 (11,27–29), which was consistent with our results. Studies assessing for IS6110 reflect a substantial portion of the literature that does not support the presence of mycobacterial DNA in the sarcoidosis tissue specimens (11,27,28,30).

A second possibility is that M. tuberculosis is present but the strains do not contain IS6110, since strains that possess one copy or no copies of IS6110 have been reported (31,32). In the United States, all of approximately 14,000 strains of M. tuberculosis tested have been shown to possess IS6110; some in low-copy number (33). Therefore, this scenario seems unlikely.

A third explanation is that while the agent we found associated with sarcoidosis has a close genetic relationship with M. tuberculosis, it is not M. tuberculosis. The genes for 16S and 23S are particularly suitable as targets for identifying microorganisms, since they are both well conserved and show variation indicative of their evolution and interrelationship with other organisms (34). This genetic variation is the basis for identifying the species of microorganisms in a particular genus, as this genetic variation is a constant property. Other members of the M. tuberculosis-complex (M. tuberculosis BCG, M. bovis, M. microti, and M. africanum) have 100% 16S and rpoB homology with M. tuberculosis but belong to different species; these strains are usually differentiated from M. tuberculosis by biochemical and clinical features. Although we could not attempt isolation of microorganisms from the formalin-fixed, paraffin-embedded specimens, future studies targeted to mycobacteria would be especially useful in confirming our observations and in characterizing any association with sarcoidosis.

We have found evidence for mycobacterial 16S rRNA and rpoB sequences in sarcoidosis tissue specimens but not in control tissue specimens. Upon sequence analysis, the products were most consistent with M. tuberculosis, but IS6110 could not be detected from these species. We also provide evidence of the presence of a heterogeneous mycobacterial population, including organisms highly related to M. tuberculosis, M. gordonae, and M kansasii. This heterogeneous population was found in individual sarcoidosis samples and, in one case, in the same sample (patient 24). These findings suggest that while M. tuberculosis and other Mycobacterium species may not be the sole microbial agents present in sarcoidosis tissues, they are commonly present and may play important roles. Further investigation into their presence and any putative etiologic agent is warranted.
